# The Flavonoid Kaempferol Ameliorates Streptozotocin-Induced Diabetes by Suppressing Hepatic Glucose Production

**DOI:** 10.3390/molecules23092338

**Published:** 2018-09-13

**Authors:** Hana Alkhalidy, Will Moore, Yao Wang, Jing Luo, Ryan P. McMillan, Wei Zhen, Kequan Zhou, Dongmin Liu

**Affiliations:** 1 Department of Human Nutrition, Foods and Exercise, College of Agricultural and Life Sciences, Virginia Tech, Blacksburg, VA 24060, USA; hkhaldi@vt.edu (H.A.); wtm247@vt.edu (W.M.); yaow@vt.edu (Y.W.); jingluo@vt.edu (J.L.); mcmillr@vt.edu (R.P.M.); weizhen@vt.edu (W.Z.); 2 Department of Nutrition and Food Technology, Faculty of Agriculture, Jordan University of Science and Technology, Irbid 22110, Jordan; 3 Department of Nutrition and Food Science, College of Liberal Arts & Sciences, Wayne State University, Detroit, MI 48202, USA; kzhou@wayne.edu

**Keywords:** kaempferol, insulin deficiency, diabetes, hepatic glucose production, gluconeogenesis, pyruvate carboxylase

## Abstract

In diabetes mellitus, the excessive rate of glucose production from the liver is considered a primary contributor for the development of hyperglycemia, in particular, fasting hyperglycemia. In this study, we investigated whether kaempferol, a flavonol present in several medicinal herbs and foods, can be used to ameliorate diabetes in an animal model of insulin deficiency and further explored the mechanism underlying the anti-diabetic effect of this flavonol. We demonstrate that oral administration of kaempferol (50 mg/kg/day) to streptozotocin-induced diabetic mice significantly improved hyperglycemia and reduced the incidence of overt diabetes from 100% to 77.8%. This outcome was accompanied by a reduction in hepatic glucose production and an increase in glucose oxidation in the muscle of the diabetic mice, whereas body weight, calorie intake, body composition, and plasma insulin and glucagon levels were not altered. Consistently, treatment with kaempferol restored hexokinase activity in the liver and skeletal muscle of diabetic mice while suppressed hepatic pyruvate carboxylase activity and gluconeogenesis. These results suggest that kaempferol may exert antidiabetic action via promoting glucose metabolism in skeletal muscle and inhibiting gluconeogenesis in the liver.

## 1. Introduction

Glucose levels are controlled by a sophisticated coordination of several pathways in both the feeding and fasting states. The increase in blood glucose levels in the portal vein post-absorption increases hepatic glucose uptake and subsequent metabolism, initiated by glucokinase (GCK) or hexokinase IV [1], and this hence increases glucose utilization and storage by the liver before it gets to the blood circulation [2]. Meanwhile, increased circulating glucose stimulates insulin secretion from pancreatic β-cells, which subsequently increases glycogen synthesis while inhibiting gluconeogenesis, thereby reducing hepatic glucose output [3,4]. In addition, muscle tissue plays a significant role in disposing blood glucose, which accounts for over one third of total disposed blood glucose as compared with about 40% by visceral organs (mostly by the liver) [5]. On the other hand, low glucose levels trigger glucagon release which simultaneously increases glucose production through hydrolysis of glycogen as well as gluconeogenesis in the liver [6,7]. Glucagon promotes the transcription of key glucogenic enzymes such as pyruvate carboxylase (PC) [8], phosphoenolpyruvate carboxykinase (PEPCK), and glucose-6-phosphatase (G6Pase) [9]. The liver is the major contributor of glucose flux into the circulation in the fasting state responsible for about 90% of the overall glucose production with the kidneys generating the rest of glucose output [10]. Besides, peripheral tissues, including the muscle and adipose tissues, provide the liver with glucogenic precursors, which are suppressed in the presence of insulin [11].

Type 1 diabetes (T1D) is an autoimmue disease that result from the immune cell-mediated destruction of pancreatic β-cells, thus causing insulin deficiency and hyperglycemia. T1D accounts for less than 10% of diabetes cases and accompanied by serious complications [12]. Like Type 2 diabetes (T2D), hepatic and peripheral glucose uptake and metabolism are also altered in T1D [13], which are associated with reduced GCK [14] and muscle hexokinase II (HK II) protein expression [15]. Additionally, it is accompanied by excessive hepatic glucose production which primarily contributes to fasting hyperglycemia, the hallmark of diabetes [16]. Due to insulin deficiency, glucagon stimulation of the key glucogenic enzymes is not restrained [17]. Indeed, the use of agents that suppress glucagon [18] or its action can prevent hyperglycemia in insulin-deficient rodent models of diabetes [19]. Despite advances in understanding T1D, there is no cure for it, and therefore patients require insulin administration for survival [20]. Continuous glucose monitoring and intensive treatment showed improvements in glycemic control of adult patients but weren’t efficient in younger patients who are more commonly diagnosed with this disease [21]. 

Kaempferol, a flavonol present in several medicinal herbs and edible plants [22], exerted many pharmacological activities in preclinical studies [23]. It has been reported that kaempferol elicits a number of health benefits, including anti-oxidative [24,25], anti-inflammatory [26,27], anti-hypertensive [28,29], lipolytic [30,31], and anti-carcinogenic effects [32,33,34]. However, limited number of studies examined the anti-diabetic properties of this compound. In the present study, we examined whether kaempferol possess anti-diabetic properties in a type 1-like diabetes, which was induced by intraperitoneal (ip) injection of multiple low doses of streptozotocin (STZ) to lean mice fed a standard chow (SD) diet [35]. Our data show that kaempferol treatment significantly ameliorated hyperglycemia in the short and long-term of treatment. Kaempferol treatment restored the activity of key enzymes, which play crucial roles in glucose metabolism such as hexokinases in the liver and muscle. Kaempferol reduced glucose output in the liver of diabetic mice, which was associated with a decrease in PC activity and glycogenolysis. These changes were independent of body weight gain, body composition, or changes in plasma insulin levels of diabetic mice. The data from this study indicate that kaempferol could be used for mitigating hyperglycemia caused by insulin deficiency.

## 2. Results

### 2.1. Kaempferol Treatment Ameliorated HyperGlycemia in Diabetic Mice

**Figure 1:**
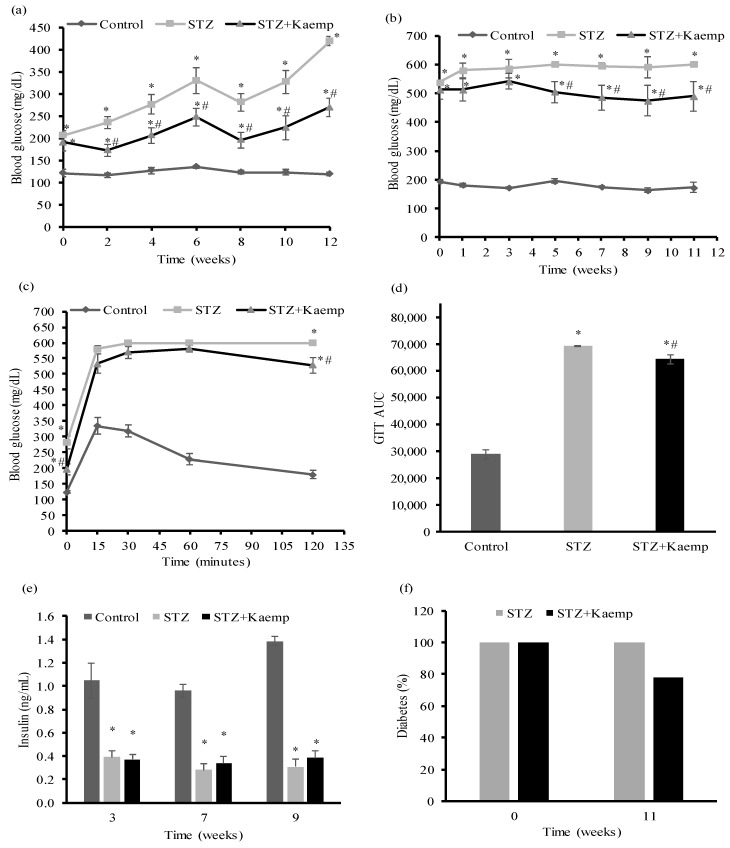
Oral administration of kaempferol improved glucose control in STZ-induced diabetic mice. (**a**) Fasting and (**b**) non-fasting blood glucose levels were measured at indicated time points during 12 weeks of kaempferol treatmnet. GTT (**c**) was performed as described in the Method section after 6 weeks of kaempferol treatment. The area under the curve (AUC) for GTT (**d**) was calculated. (**e**) Non-fasting blood was withdrawn at week 3, 7, and 9 after treatment for measuring plasma insulin levels. (**f**) The percentage of mice with non-fasting blood glucose levels of >300 mg/dL was calculated at the beginning and after 11 weeks of kaempferol treatment. Data are shown as Mean ± SEM (*n* = 9). * *p* < 0.05 vs. non-diabetic mice; # vs. STZ-induced diabetic mice. Control: nondiabetic mice; STZ: STZ-induced diabetic mice; STZ + Kaemp: STZ-induced diabetic mice treated with kaempferol (50 mg/kg BW).

To determine whether kaempferol is capable of reversing or ameliorating hyperglycemia following overt diabetes, we generated insulin deficient diabetic mice by injecting multiple low doses of STZ [36]. Mice with non-fasting blood glucose levels >350 mg/dL after 3 weeks post-STZ administration were then treated with kaempferol or vehicle for 12 weeks. Kaempferol treatment significantly reduced both fasting (Figure 1a) and non-fasting (Figure 1b) blood glucose levels of diabetic mice. Consistently, STZ-induced diabetic mice displayed severe glucose intolerance during an intraperitoneal glucose tolerance test, which was ameliorated by kaempferol treatment (Figure 1c,d). While diabetic mice had significantly lower non-fasting plasma insulin levels than those in non-diabetic mice, kaempferol had no effect on the circulating insulin levels in the diabetic mice during the treatment period (Figure 1e), suggesting that better glycemic control elicited by kaempferol treatment is not related to any improvements of the insulin secretory function of pancreatic islets. Previous studies showed that mice with non-fasting blood glucose levels above 300 mg/dL are considered diabetic [37]. Based on this diagnostic threshold, we calculated the percentage of diabetes before and 12 weeks after kaempferol treatment. We found that kaempferol reduced the incidence of overt diabetes from 100% to 77.8%, whereas the percentage of diabetic mice in the control group remained at 100% with glucose levels of greater than 400 mg/dL (Figure 1f). 

**Figure 2:**
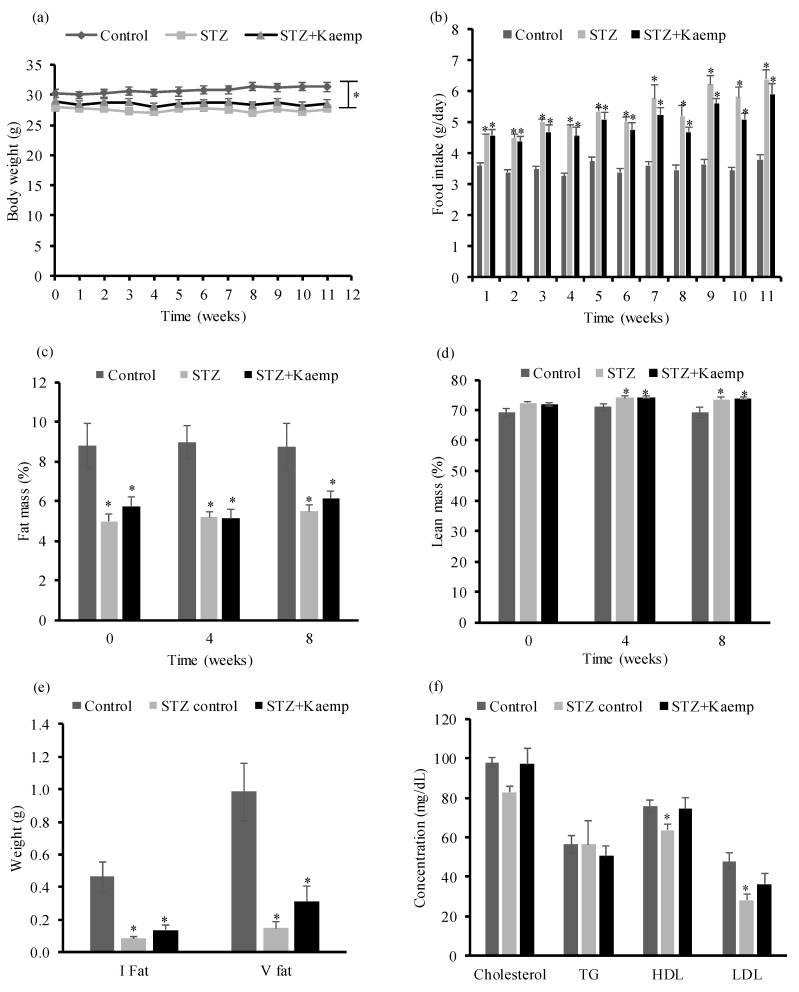
Kaempferol treatment for 12 weeks did not alter BW, calorie intake, or body composition, but restored lipid profiles in STZ-induced diabetic mice. (**a**) BW of the individual mouse was measured weekly. (**b**) Food intake was recorded weekly, and the average daily food intake was calculated. Body composition including fat mass (**c**), and lean mass (**d**) were measured at 0, 4, and 8 weeks of treatment. At the end of the study, (**e**) inguinal and visceral fat were weighed and (**f**) plasma lipid profile were analyzed. Data are shown as Mean ± SEM (*n* = 9). * *p* < 0.05 vs. Control nondiabetic mice. Control: nondiabetic mice; STZ: STZ-induced diabetic mice; STZ + Kaemp: STZ-induced diabetic mice treated with kaempferol (50 mg/kg BW).

However, oral provision of kaempferol did not exert any effect on body weight (BW) (Figure 2a), food intake (Figure 2b), fat mass (Figure 2c) or lean mass (Figure 2d). Consistently, the inguinal and visceral fat contents were significantly lower in diabetic mice as compared with those in nondiabetic control mice, and kaempferol had no effect on these parameters (Figure 2e). To assess the effect of kaempferol treatment on lipid profiles, we measured the concentrations of plasma lipids. We observed that untreated diabetic mice had lower total cholesterol, HDL-cholesterol (*p* < 0.05), and LDL-cholesterol (*p* < 0.05) levels when compared to those in non-diabetic mice. Kaempferol treatment reversed these changes to the levels similar to those seen in non-diabetic mice, but triglycerides levels were not different between all groups (Figure 2f).

**Figure 3:**
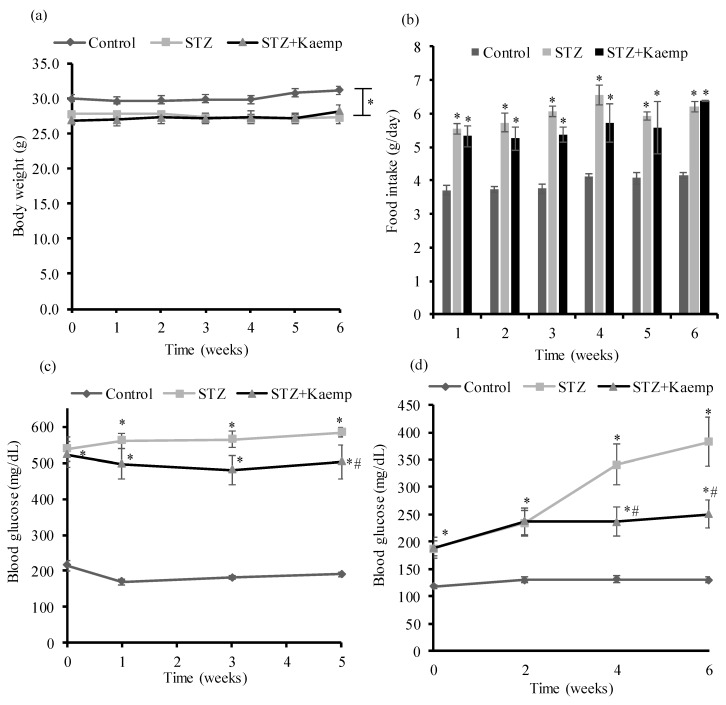
Kaempferol treatment for 6 weeks improved hyperglycemia without altering BW or food intake in STZ-induced severely diabetic mice. (**a**) BW was measured every week. (**b**) Food intake was measured weekly and are expressed as daily food intake. (**c**) Non-fasting and (**d**) fasting blood glucose levels were measured at indicated time points of dietary treatment. Data are shown as Mean ± SEM (*n* = 8–9). * *p* < 0.05 vs. non-diabetic mice; # vs. STZ-induced diabetic mice. Control: nondiabetic mice; STZ: STZ-induced diabetic mice; STZ + Kaemp: STZ-induced diabetic mice treated with kaempferol (50 mg/kg BW).

To confirm the anti-diabetic effect of kaempferol, a second study was performed to further evaluate whether kaempferol treatment is still effective when mice are severely diabetic. In that regard, mice became overt diabetic for 6 weeks with non-fasting blood glucose levels over 500 mg/dL before initiating treatment with kaempferol. Consistently, we observed that kaempferol did not affect BW or food intake during the time course of the study (Figure 3a,b). Consistently, kaempferol treatment for 1 week already ameliorated hyperglycemia in the non-fasting state (Figure 3c), similar to the observation in the first animal study. However, it took longer time (4 weeks of treatment vs. 2 weeks in the first study) for kaempferol to ameliorate fasting blood glucose (Figure 3d). These findings confirmed that kaempferol is effective in ameliorating hyperglycemia, and it is more effective in ameliorating hyperglycemia in mice if the treatment started at the earlier stage of diabetes. 

### 2.2. Kaempferol Suppressed Hepatic Gluconeogenesis

**Figure 4:**
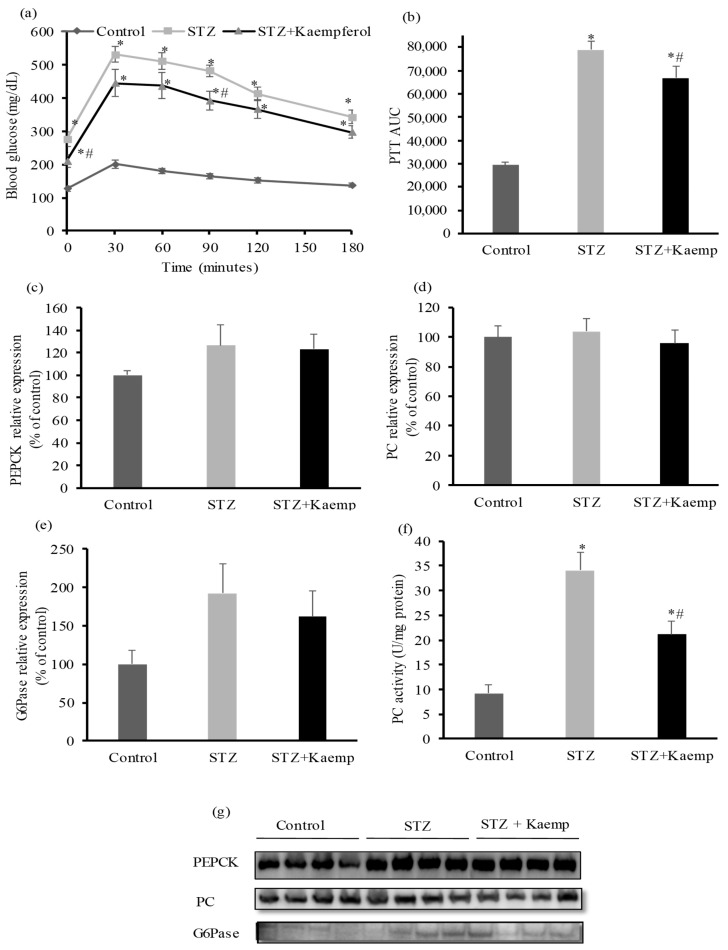
Kaempferol treatment for 12 weeks decreased hepatic glucose production from pyruvate and reduced PC activity without altering protein expressions of PC, PEPCK, or G6Pase in STZ-induced diabetic mice. PTT (**a**) was performed as described in the Method section, and the area under the curve (AUC) of PTT (**b**) was calculated. (**c**,**g**) PEPCK, (**d**,**g**) PC, and (**e**,**g**) G6Pase protein levels in the livers were measured by Western blot normalized to total protein contents in whole cell lysates. (**f**) PC activity was measured as described in the Method section. Data are shown as Mean ± SEM (*n* = 8–9). * *p* < 0.05 vs. non-diabetic mice; # vs. STZ-induced diabetic mice. Control: nondiabetic mice; STZ: STZ-induced diabetic mice; STZ + Kaemp: STZ-induced diabetic mice treated with kaempferol (50 mg/kg BW).

**Figure 5:**
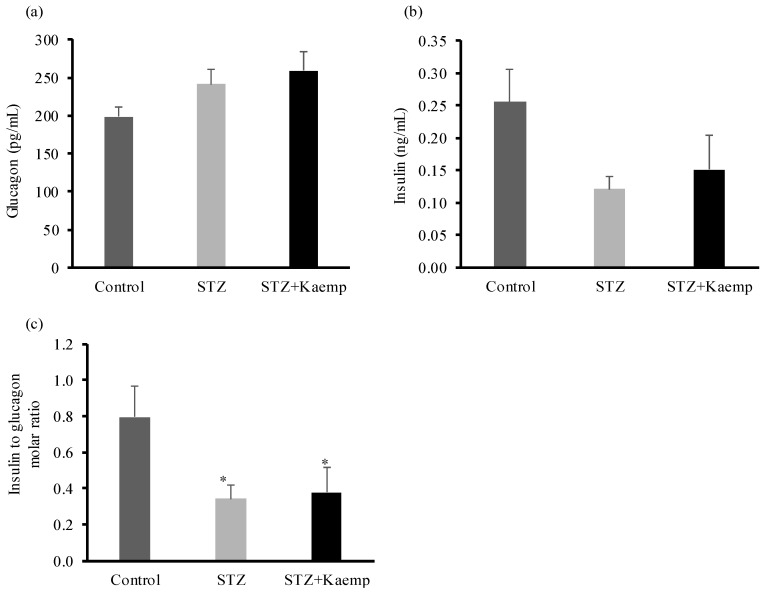
Kaempferol had no effect on the concentrations of circulating plasma insulin or glucagon levels. After 12 weeks of treatment with kaempferol, (**a**) glucagon, and (**b**) insulin levels in the blood were measured using ELISA kits, and (**c**) their molar ratio was calculated. Data are shown as Mean ± SEM (*n* = 8–9). * *p* < 0.05 vs. Control nondiabetic mice. Control: nondiabetic mice; STZ: STZ-induced diabetic mice; STZ + Kaemp: STZ-induced diabetic mice treated with kaempferol (50 mg/kg BW).

To determine the anti-diabetic mechanism of kaempferol in mice, we first evaluated gluconeogenesis by performing pyruvate tolerance test. Hepatic glucose production increased (*p* < 0.05) in diabetic mice as compared to non-diabetic mice [38]. As shown in Figure 4a,b, kaempferol treatment suppressed hepatic gluconeogenesis (*p* < 0.05), thereby glucose output in diabetic mice. Next, we measured fasting circulating glucagon and insulin levels and calculated insulin to glucagon molar ratio. There were no significant differences in glucagon or insulin levels between treated and control diabetic mice. Also, the ratio of these two hormones was not altered by kaempferol treatment (Figure 5a–c). Further, we examined the key enzymes that regulate gluconeogenesis in the liver including PC, PEPCK, and G6Pase. Kaempferol treatment had no effect on PEPCK (Figure 4c,g), PC (Figure 4d,g), or G6Pase (Figure 4e,g) protein levels in diabetic mice. Diabetic mice displayed significantly higher PC activity as compared with healthy mice, but kaempferol significantly suppressed the elevated PC activity in diabetic mice (Figure 4f). These results suggest that kaempferol improves glycemic control in diabetic mice at least in part through suppressing gluconeogenesis in the liver, and further suggest that kaempferol may inhibit gluconeogenesis via regulating pyruvate carboxylation, the first and critical step in gluconeogenesis. 

### 2.3. Kaempferol Increased GCK Activity and Glycogen Content in the l-Liver without Affecting Glucose Oxidation

**Figure 6:**
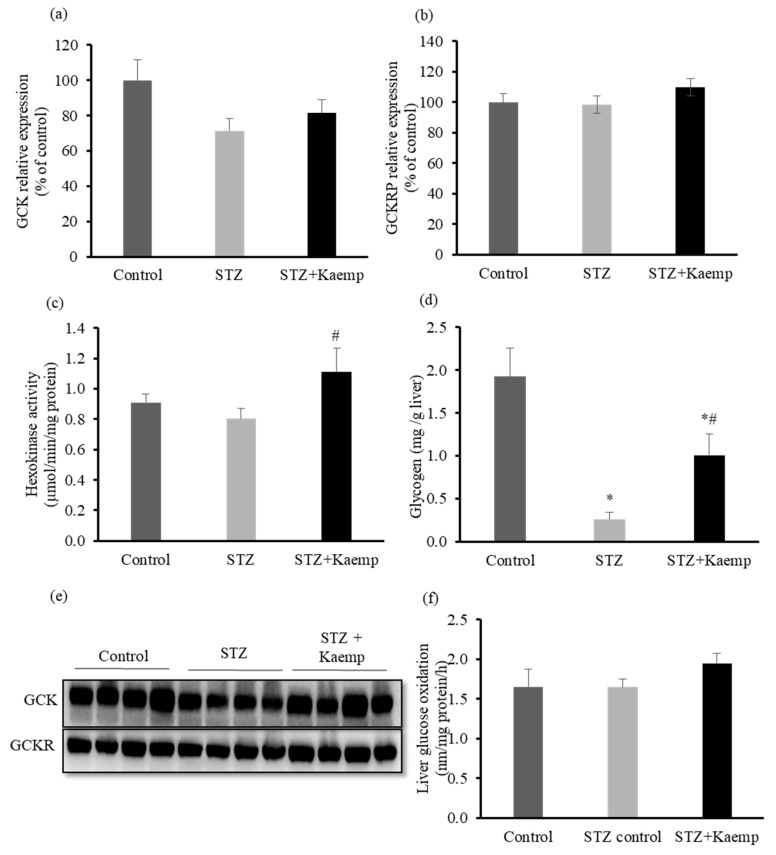
Kaempferol increased GCK activity and glycogen contents in STZ-induced diabetic mice. Diabetic mice were treated with kaempferol for 12 weeks. (**a**,**e**) Hepatic GCK, and (**b**,**e**) GCKRP protein levels were measured by Western blot and normalized to total protein contents. Hexokinase activity (**c**) and glycogen content (**d**) in the liver were measured as described in the Method section. (**f**) Glucose oxidation was evaluated in fresh mouse liver homogenates using ^14^C-labeled glucose as described in the Method section. Data are shown as Mean ± SEM (*n* = 8–9). * *p* < 0.05 vs. non-diabetic mice; # vs. STZ-induced diabetic mice. Control: nondiabetic mice; STZ: STZ-induced diabetic mice; STZ + Kaemp: STZ-induced diabetic mice treated with kaempferol (50 mg/kg BW).

In insulin-deficient mice, GCK expression in the liver decreases, which impairs hepatic glucose uptake and utilization [14]. We found that diabetic mice had lower GCK protein levels when compared to non-diabetic mice, while glucokinase regulatory protein (GCKRP) protein levels was similar between groups. Kaempferol treatment restored GCK activity (*p* < 0.05), whereas it had no effect on either GCK or GCKRP protein expression in diabetic mice (Figure 6a–c,e). This increase in the activity of GCK in the liver can direct more glucose into glycogen synthesis and indirectly reduces the overall glucose flux from the liver [1,2]. To determine whether kaempferol decreases glycogenolysis, thereby contributing to the reduced glucose output as noticed in kaempferol-treated mice, we measured glycogen contents in the liver. 

As shown in Figure 6d, diabetic mice had lower glycogen contents (*p* < 0.05) as compared with the non-diabetic mice. However, treatment with kaempferol partially restored glycogen contents (*p* < 0.05) in diabetic mice (Figure 6d). To examine whether kaempferol may have any effect on channeling glucose to other destinations, we measured glucose oxidation in the liver. There was no effect of kaempferol treatment on glucose oxidation in the liver (Figure 6f). 

### 2.4. Kaempferol Increased Hexokinase Activity and Glucose Oxidation in Red Muscle

**Figure 7:**
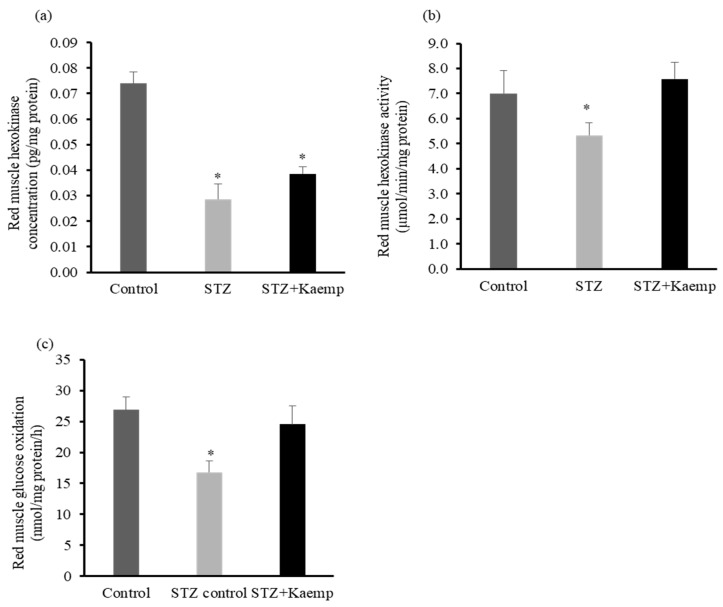
Kaempferol increased hexokinase activity and glucose oxidation in red skeletal muscle of STZ-induced diabetic mice. Following 12 weeks of treatment with kaempferol, (**a**) mouse liver hexokinase concentration and (**b**) activity were measured and normalized to total protein contents in the cell lysates. (**c**) Glucose oxidation in fresh mouse muscle homogenates was assayed using 14C-labeled glucose as described in the Method section. Data are shown as Mean ± SEM (*n* = 8–9). * *p* < 0.05 vs. Control nondiabetic mice. Control: nondiabetic mice; STZ: STZ-induced diabetic mice; STZ + Kaemp: STZ-induced diabetic mice treated with kaempferol (50 mg/kg BW).

Insulin stimulates the transport and uptake of glucose [39] and consequently increases hexokinase activity in muscle [40]. Activation of hexokinase induces glucose phosphorylation that subsequently triggers glycolysis and glycogen synthetic processes [41]. Therefore, insulin deficiency reduces muscle glucose uptake as well as glucose metabolism. To examine whether kaempferol affected glucose metabolism in skeletal muscle, we measured hexokinase protein level and activity as well as glucose oxidation. Diabetic mice had significantly lower hexokinase protein levels as compared with non-diabetic mice. Consistent with the observation in the liver, kaempferol restored the reduced hexokinase activity to the level comparable to that in nondiabetic mice (Figure 7a,b).

In addition, kaempferol increased glucose oxidation in skeletal muscle (*p* < 0.05) (Figure 7c), which could be the secondary action whereby kaempferol treatment improved hexokinase activity. These findings suggest that kaempferol enhanced glucose metabolism in skeletal muscle of the diabetic mice.

## 3. Discussion

In this study, we demonstrate that a single daily dose of kaempferol via gavage can significantly ameliorate hyperglycemia and enhance glucose tolerance in insulin deficient mice, which is associated with increased glucose disposal and utilization, in particular, oxidation in muscle tissue and suppressed hepatic glucose production. These favorable outcomes are mediated by normalizing the activity of the regulatory enzymes involved in controlling glucose metabolism and homeostasis. These effects of kaempferol were independent of insulin or glucagon concentrations and were not accompanied by changes in BW gain, food intake, or body composition. In both T1D and T2D, dysregulated glucose metabolism characterized by the combination of reduced glucose utilization and increased hepatic glucose production plays an important role in the deterioration of blood glucose control. Thus, kaempferol could be used as an adjuvant treatment for maintaining glucose homeostasis by targeting the glucose production and metabolic pathways.

The reciprocal relationship between the hormones glucagon and insulin in the regulation of glucose homeostasis is disrupted in STZ-induced diabetic mice due to the destruction of β-cells [42]. Therefore, the amount of insulin secreted from the residual β-cells is insufficient to oppose glucagon action on hepatic glucose production or to inhibit its secretion, which results in excessive glucose production [43,44]. In the present study, it is evident that kaempferol treatment lowered blood glucose levels both in the fasting and non-fasting states without altering insulin or glucagon concentrations in diabetic mice. However, after 11 weeks of treatment with kaempferol, diabetic mice remained hyperglycemic, suggesting that kaempferol is not sufficient in reversing diabetes in this animal model. The improved glucose control in kaempferol-treated mice can be partially explained by the increase in the activity of hexokinases in the liver and skeletal muscle due to their significant role in glucose disposal and metabolism. While insulin promotes the transcription of the major hexokinase in the liver, GCK, glucagon inhibits it [45]. Although our results show that kaempferol non-significantly increased GCK protein levels, which were still lower than those in non-diabetic mice, its activity was completely restored by kaempferol treatment. However, it is still unclear how kaempferol increased GCK activity. 

GCK activity is regulated by a specific protein, GCKRP, which sequesters GCK in the nucleus in its inactive form during fasting while releasing it to the cytoplasm when glucose supply increases [46]. While we found that kaempferol did not alter GCKRP protein expression, it could modulate GCKRP-GCK interaction, thereby affecting its cytoplasmic translocation. It was reported that some flavonoids are potent inhibitors of p300 acetyltransferase (HAT) activity, thereby suppressing the acetylation of its targeted proteins [47,48]. Interestingly, recent studies showed that GCKRP in hepatocytes can be acetylated by p300 HAT, leading to the inhibition of GCKRP-GCK dissociation and thereby GCK nuclear export [49,50]. In the livers of obese diabetic mice, it was found that both p300 HAT activity [51] and GKRP acetylation levels [49] were elevated, and inhibition of p300 HAT improved insulin sensitivity and glucose control [51]. Consistently, deacetylation of GCKRP dissociates it from GCK, leading to improved hepatic glucose metabolism and glucose tolerance in obese diabetic mice [49,50]. These results underscore an important role for p300 HAT in dysregulated hepatic glucose metabolism mediated via acetylation GCKRP. Therefore, it is intriguing to speculate that kaempferol may increase GCK activity through inhibition of p300 HAT-mediated GCKRP acetylation, which could occurs via hydrophobic interaction between its aromatic moiety and the enzyme [52].

It is well established that glucose disposal is facilitated by the combined influence of both glucose and insulin [53]. In the muscle tissue, insulin was reported to stimulate glucose transport and phosphorylation [54], which is then routed to different destinies; oxidation, glycogen synthesis, or glycolysis pathway [55,56]. Insulin can also increase the activity of muscle hexokinase through increasing its protein level and modifying the already existing kinase [40]. In the present study, hexokinase levels and activity, as well as glucose oxidation, were decreased in the muscle of insulin-deficient diabetic mice. However, kaempferol restored hexokinase function and increased the rate of glucose oxidation. The increase in glucose oxidation observed in skeletal muscle of kaempferol-treated mice might be secondary whereby kaempferol increased hexokinase activity, which in turn stimulates glycolysis [41]. Further, it has been shown that the increase in hexokinase activity is accompanied with an increase in the membrane translocation of the primary glucose transporter (GLUT4) [57], which subsequently increases glucose uptake [58], suggesting that kaempferol may increase glucose uptake and utilization via modulating this pathway. However, studies are still needed to decide whether these molecules are directly targeted by kaempferol.

T1D is associated with lipoprotein abnormalities [59]. Although the abnormalities may vary, it is documented that T1D subjects may experience normal to low levels of blood lipoprotein concentrations compared to healthy subjects [60]. Similar findings were observed in SD-fed STZ-induced diabetic mice [61]. Here we show that kaempferol reversed the abnormalities of LDL and HDL concentrations that might be due to improvements in glucose control [62]. Nevertheless, the defects in composition and size of lipoproteins, and in particular LDL [63] may lead to atherosclerosis in diabetic subjects [59,60] and experimental animals [61]. Thus, experiments may be necessary to help understand how kaempferol improves lipoprotein synthesis and metabolism.

Excessive glucose production contributes significantly to hyperglycemia in experimental diabetes [64] and T1D subjects [16]. PC, a mitochondrial enzyme, catalyzes the reaction that produces oxaloacetate from pyruvate, the initial and critical step in gluconeogenesis [65]. While the functions and regulation of PC are still under investigation [66], its crucial role in the control of gluconeogenesis is well-established [8,67,68]. Recently, it has been proposed that reducing PC activity in the liver is a potential target for reducing hepatic glucose production from gluconeogenesis [69]. Therefore, the anti-diabetic action of kaempferol observed in the present study is at least partially ascribed to its suppressing action on hepatic glucose production, as examined by performing the pyruvate tolerance test. This effect of kaempferol might be mediated via inhibiting the activity of PC. Additionally, increased liver glycogen content, which could be due to the reduced glycogenolysis, may also be a contributor to the overall decreased glucose output detected in mice treated with kaempferol. However, the mechanism by which kaempferol reduces PC activity and glycogenolysis in the liver is currently unknown. Further research is needed to study these pathways.

In summary, we provide evidence that oral provision of kaempferol improved glucose control in insulin-deficient diabetic mice. This effect of kaempferol was accompanied by a reduction in hepatic gluconeogenesis and improved metabolism in the liver and skeletal muscle. These results indicate that kaempferol, which is naturally present in several plants, may be an anti-diabetic agent to be used as an adjuvant treatment for diabetes. More research is needed to elucidate the underlying mechanism by which kaempferol regulates gluconeogenesis and glucose metabolism.

## 4. Methods

### 4.1. Mice, STZ Administration, and Kaempferol Treatment

Male (5.5 mo old) C57BL/6 male mice (Envigo, Indianapolis, IN, USA) were kept on a 12-h light/dark cycle at constant temperature (22–25 °C) and have *ad libitum* access to a standard rodent chow (SD) diet and water. All experimental protocols were approved by the Institutional Animal Care and Use Committee at Virginia Tech (protocol#: IACUC-14-197). For the first study, diabetic mouse model was developed with intraperitoneal injection of STZ (40 mg/kg BW) for 4 consecutive days. Control mice were given the same amount of vehicle (10 mM sodium citrate, pH 4.5). Three weeks after STZ injection, mice with sustained hyperglycemia of >350 mg/dL were divided into 2 groups (*n* = 9 mice/group), with non-fasting blood glucose levels, BW, and body composition balanced among groups, and then given either kaempferol (50 mg/kg/day dissolved in 2% 2-methyl cellulose) or vehicle by oral gavage for 12 weeks. Nondiabetic mice (*n* = 9 mice) served as a control group and received the vehicle. For second animal study, STZ-induced diabetic mice (C57BL/6 male) were divided into 2 groups (*n* = 8–9 mice/group). Six weeks after STZ injection, mice were administered kaempferol (50 mg/kg/day dissolved in 2% 2-methyl cellulose) or vehicle by oral gavage for 7 weeks. 

### 4.2. Metabolic Studies

BW and food intake were measured every week through the study. Blood glucose levels were measured biweekly using a glucometer (Kroger, Cincinnati, OH, USA). Body composition of the mice was examined at 0, 4, and 8 weeks after treatment using an LF-90 instrument (Bruker Optics, Inc., Billerica, MA, USA). Four and 6 weeks after treatment with kaempferol, mice fasted for 15 h were administered via i.p., injection a single dose of pyruvate (1 g/kg BW), or glucose (1 g/kg BW) for pyruvate and glucose tolerance tests, respectively. Blood glucose levels were then measured at 0, 30, 60, 90, and 180 min and 0, 15, 30, 60, and 120 min after administration of pyruvate or glucose. The area under the curve (AUC) for these tests was calculated using the trapezoidal rule [70]. At the end of the study, the mice were fasted for 15 h and were then euthanized between 9–11 a.m. Blood was collected immediately, and various organs were weighed and snap-frozen in liquid nitrogen and then stored at −80 °C for further analyses. Plasma lipid profile was analyzed by enzymatic methods using assay kits (Teco Diagnostics, Anaheim, CA, USA). Plasma insulin levels were measured using an ultrasensitive mouse insulin ELISA kit (Mercodia, Inc., Uppsala, Sweden). Plasma glucagon levels were measured using mouse glucagon ELISA kit (Crystal Chem, Downers Grove, IL, USA).

### 4.3. Glucose Oxidation Assay

Fresh mouse liver and muscle (red) samples were used to analyze glucose oxidation as previously described [71,72] with modifications. Briefly, liver or muscle tissues were homogenized in the buffer (0.250 M Sucrose, 1 mM EDTA, 0.01 M Tris-HCl, and 2 mM ATP, pH = 7.4). The tissue homogenates were then incubated with ^14^C-labeled glucose (American Radiolabeled Chemicals, St. Louis, MO, USA) at 37 °C for 1 h. The ^14^CO_2_ generated was trapped with 70% perchloric acid and the resulting sodium hydroxide was then used to assess CO_2_ production. 

### 4.4. Enzyme Activity Assays

Enzymatic activity of kexokinase in the liver and muscle tissues were measured using an assay kit (Biomedical Research Services Center, Buffalo, NY, USA) as described [73]. The activity of hexokinase in the cell lysates was calculated using this equation: IU/L unit= μM/(L·min) = (O.D. × 1000 × 110 µL/(30 min × 0.6 cm× 18 × 10 µL). Pyruvate carboxylase activity was measured using a citrate synthase-coupled reaction as previously described [74].

### 4.5. Glycogen Content Measurement

Mouse liver was homogenized in phosphate buffer (pH 7.0), centrifuged (9500 rpm for 10 min at 4 °C), and the supernatants were then collected for measuring glycogen concentrations using a kit (Cayman, Ann Arbor, MI, USA). 

### 4.6. Western Blot Analysis

Tissue lysates were resolved on 10% SDS-PAGE gels, blotted onto nitrocellulose membranes, and then probed with antibody against PEPCK (H-300), G6Pase (H-60), PC (H-300), GCK (H-88), or GCK regulatory protein (N-19) (Santa Cruz Biotechnology, Inc., Dallas, TX, USA) as we previously described [75]. The immune-reactive proteins were detected by chemiluminescence and quantified using ChemiDoc™ Touch Imaging System (Bio-Rad, Hercules, CA, USA). The protein levels were normalized to those of total protein in the same lane.

### 4.7. Statistical Analysis

Data were analyzed with one-way ANOVA using SigmaPlot^®^ software (Systat Software Inc., San Jose, CA, USA). Treatment differences were subjected to Duncan’s multiple range test, and a *p*-value < 0.05 was considered significant. Values are expressed as mean ± standard error of mean (SEM).
